# Predicting pregnancy rate and live birth rate in the IVF clinic by analysing patient profiles

**DOI:** 10.1186/2049-3258-73-S1-P46

**Published:** 2015-09-17

**Authors:** Shangshang Wang, Geert Molenberghs, Carl Spiessens, Diane De Neubourg, Adelheid Soubry

**Affiliations:** 1 Leuven Statistics Research Centre, Leuven, Belgium; 2 Interuniversity Institute for Biostatistics and Statistical Bioinformatics, KUL, Leuven, Belgium; 3 Leuven University Fertility Center, UZ Leuven Campus Gasthuisberg, Leuven, Belgium; 4 Epidemiology Research Group, Dept. of Public Health and Primary Care, KU Leuven, Leuven, Belgium

## 

This study used data of *in vitro* fertilization (IVF) cycles from 3,221 patients during 2004 to 2013, collected in Leuven University Fertility Center, aiming to identify patient and cycle characteristics to predict pregnancy and live birth rate. Variables include age, gonadotrophin dose, year of the IVF cycle, implantation problems, transport problems, ovulation problems, male pathology, pituitary inhibition, treatment, medication, and endometriosis status.

One major issue of our dataset was the presence of missing data and unknown' fill-in in some of the variables. Due to the conceptual overlapping between missing and unknown', we employed two different setups to treat the unknowns': 1) as a new category; 2) as missing values. As ignoring the missingness would have significant impact on potential conclusions, we conducted multiple imputation on the missing data. The logistic regression model inclusive of a subject-specific random intercept was next fitted to the imputed dataset to assess the effect of the variables on the probability of transfer/pregnancy success. A sensitivity analysis was carried out to check for the influence of missing mechanism. For the prediction purpose, the non-parametric Random Forest model was used, and 10-fold cross-validation was performed to obtain the prediction accuracy.

Our results suggest that the outputs of these two setups are mostly comparable in the generalized linear mixed model. Other than the most well-known factor-age, we found that gonadotrophin dose, transport problems, ovulation problems, pituitary inhibition also have significant main effects on pregnancy rate. The same set of variables plus implantation problems and treatment have significant fixed effect on live birth rate. The random forest model can achieve average prediction accuracy of 53% on transfer outcome and 52% on pregnancy outcome, which indicates that our current set of variables are not enough to yield good prediction performance on IVF outcomes.

